# The influence of workload on muscle fatigue, tissue properties, and postural stability in older and younger workers

**DOI:** 10.1371/journal.pone.0316678

**Published:** 2025-01-03

**Authors:** Julien Ducas, Janny Mathieu, Michael Drouin, Stéphane Sobczak, Jacques Abboud, Martin Descarreaux

**Affiliations:** 1 Department of Human Kinetics, Université du Québec à Trois-Rivières, Trois-Rivières, Québec, Canada; 2 Groupe de Recherche sur les Affections Neuromusculosquelettiques (GRAN), Université du Québec à Trois-Rivières, Trois-Rivières, Québec, Canada; 3 Department of Anatomy, Université du Québec à Trois-Rivières, Trois-Rivières, Québec, Canada; Martin-Luther-University Halle-Wittenberg, GERMANY

## Abstract

Demographic aging and extended working lives have prompted interest in the physiological changes that occur with age, particularly in the lumbar spine. Age-related declines in muscle quality and intervertebral disc alterations may reduce muscular endurance, strength, and postural stability, potentially increasing the risk of musculoskeletal injuries in older workers. As experienced workers play an important role in addressing labor shortages, understanding the impact of age-related physiological changes on the biomechanical properties of the lumbar spine is key to ensure safe and sustainable employment for aging individuals. This study aimed to compare the impact of daily work-related physical efforts on lumbar muscular endurance and fatigue, spine tissue properties, and postural stability between older and younger workers. A total of 40 participants, 20 in Group 1 (young workers: ≤50 years; mean age: 28.89 ± 7.23) and 20 in Group 2 (older workers: >50 years; mean age: 59.40 ± 5.29) were recruited. Measurements taken at the beginning and end of the workday included lumbar muscle endurance, maximal voluntary contraction, disc height and postural stability. Age groups were compared using repeated measures ANOVA across the two measurement times. No significant interaction between age and time of day was observed, indicating that, for similar workload, both age groups experienced similar changes. Despite age-related effects on maximal force production and postural stability, incorporating weight as a covariate revealed that these differences were partially explained by the weight discrepancy between older and younger workers. The study suggests that age may not be the primary determinant of the impact of a workday on older workers.

## Introduction

Western countries are currently facing numerous challenges associated with demographic aging. A noteworthy trend in recent years has been the extension of the working lives of individuals, particularly those aged between 55 and 64 years [1–3]. In the last decade, the economic context has prompted governments to promote public policies aiming to extend working lives, moving away from encouraging early retirement to, nowadays, offering financial incentives to older-aged employers and assisting reentry of older workers into the workforce [3]. Experienced workers are now seen as part of the solution to labor shortages, as they are showing increasing interest in remaining active in the workforce [3, 4].

Physiological changes resulting from aging may manifest in various forms and severity. For instance, while all individuals experience muscle mass decline after the age of 50, not all elderly people suffer from sarcopenia (i.e., a severe loss of muscle mass) [5]. Structural changes in muscle quality (i.e., decreased fiber length and volume, substitution of fast-twitch type II fibers with slow-twitch type I fibers and increased intramuscular fat) are known to impact muscle function, even in healthy aging individuals [6, 7]. Age-related alterations in muscle volume and fat fraction have been observed in the lumbar paravertebral muscles [7], potentially leading to decreased muscular strength, endurance and postural stability [6, 8, 9]. Additionally, the intervertebral disc (IVD) undergoes age-related histomorphological and functional alterations [10]. These changes can alter the biomechanical properties of the IVD and increased susceptibility to musculoskeletal injuries [10–12]. The histomorphological and functional changes affecting the IVD begin as early as the second decade of life, with increasing prevalence with advancing age [10, 13]. Studies suggest that alterations in the biomechanical properties of the IVD and changes in muscle quality are interconnected [10, 14]. Indeed, statistically significant negative correlations have been found between the severity of disc herniation and muscle volume [14, 15].

Professional activities involving prolonged sitting or standing, as well as repetitive lifting, can have significant effects on neuromuscular function, particularly in the lumbar spine structures. For instance, sitting or standing for an extended period can induce muscle fatigue and increase the relative contribution of lumbar passive tissues (i.e., ligaments, joint capsules, discs) in maintaining an upright torso [16, 17]. Despite the growing number of aging workers delaying retirement, there is limited evidence on the impact of age-related physiological changes on the ability to perform work activities. The few studies available suggest that prolonged work, simulated in research settings, results in muscle fatigue, and increased postural oscillations in both young and older workers [18, 19]. However, most studies investigating the impact of the workload on neuromuscular function and on postural stability in aging workers have focused on the lower limb [8, 18–20]. Consequently, further studies are needed to better understand the impact of physiological changes resulting from aging, specifically in the lumbar spine, to ultimately implement strategies that will promote health maintenance and extended working life.

Therefore, the aim of this study was to determine the impact of daily work-related physical efforts on lumbar muscular endurance and fatigue, IVD property, and postural stability in older workers, and to compare these impacts with those observed in younger workers. It was hypothesized that, for a similar workload, older workers would undergo more considerable physiological and biomechanical changes during a workday compared to younger workers.

## Materials and methods

### General study procedure

Two groups of workers were recruited: an older group of workers aged 50 and above, and a group of younger workers (≤50 years). Both groups were tested before and after a workday. During these visits, participants completed evaluations of physical and physiological variables, as well as objective self-reported measures. The physical and physiological evaluations included (1) a back extensor task to assess muscle fatigue using the modified Sorensen task, (2) a spine height assessment using a stadiometer, and (3) a postural stability test conducted on a force platform. The objective self-reported measures included sociodemographic characteristics and job satisfaction. Low job satisfaction is associated with an increased incidence of low back pain [21], which should therefore be considered as a potentially confounding variable in the present study. A study by Golabadi et al. demonstrated that high job demands, coupled with low job control and satisfaction significantly increased the incidence of back symptoms among nursing personnel [22].

### Participants

A total of 40 workers with diverse job titles as defined by the *Secrétariat du Conseil du Trésor du Québec* (e.g. laborer, administrative and teaching positions) [23] were recruited from January 15, 2020, to July 12, 2023. A total of 39 participants was required to achieve a statistical power of 0.8 and an alpha of 0.05 for a repeated measures ANOVA (2x2, disc height variable) with a moderate effect size, as calculated using G*Power 3.1 [24], while accounting for a 15% dropout rate. To ensure equal group sizes, we recruited 40 participants. Whenever possible, participants in Group 1 were matched with a participant in Group 2 with a similar job type, to minimize the impact of job characteristics. Participants were recruited from various workplaces in the Québec region. A member of the research team contacted department heads from different organizations to discuss the project and invite workers to participate. Participants had to meet the following inclusion criteria: being actively employed (full or part-time), aged 18 or older and participants needed to spend less than 50% of their workday in a seated position. Hours spent seated were self-reported by participants and subsequently validated with the employer. The exclusion criteria included taking medical leave in the three months preceding the assessment, having physical limitations hindering task performance, suffering from an active disabling pathology in the spine or lower limbs, and having an active file with the *Commission des normes*, *de l’équité*, *de la santé et de la sécurité du travail* (workers’ compensation board). Approval for the project was obtained from the Research Ethics Board for human research at "Université du Québec à Trois-Rivières" (CER-19-263-07.01), and all participants provided written informed consent. The study was conducted in accordance with the principles outlined in the Declaration of Helsinki (2013). Job satisfaction questionnaire was assessed using the Minnesota Satisfaction Questionnaire [25, 26]. The French validated version was used [26]. The short version of this questionnaire assesses 20 items detailing intrinsic satisfaction (related to individual factors), extrinsic satisfaction (related to environmental factors), and overall job satisfaction. Both intrinsic and extrinsic satisfaction subscales show high levels of internal consistency (intrinsic: α = 0.8756; extrinsic: α = 0.7796) [26]. Results can be interpreted in percentiles compared to normative groups for different types of jobs. A score above the 75^th^ percentile indicates high satisfaction, while a score below the 25th percentile indicates low satisfaction.

### Muscle fatigue

To assess muscle fatigue, two tests were conducted, a maximal voluntary isometric back extension contraction (MVIC) and a submaximal endurance task. Three indicators of muscle fatigue were measured during these tasks: [1] peak force measures during the MVIC, [2] the time to task failure during the submaximal endurance task, and [3] electromyographic (EMG) measurements during the endurance task. To perform these tasks, the modified Sorensen test was used as it allows for the measurement of lumbar muscle fatigue [27, 28]. The modified Sorensen test involved lying prone with their upper body positioned horizontally to the ground. Their hips were flexed, resting on a surface inclined at a 45-degree angle. The upper edge of the participants’ iliac crests was aligned with the edge of the table to ensure proper hip support during the test. Two straps were used to immobilize and stabilize the lower limbs, placed at the pelvic and ankle levels. Initially, force measurements were conducted by having participants exert their MVIC force against a shoulder-attached belt for five seconds. The belt was connected to a force gauge (Model LSB350; Futek Advanced Sensor Technology Inc, Irvine, CA, USA). Three MVIC trials were performed with one-minute rest intervals to minimize fatigue, and a five-minute rest was provided between tasks. The highest force value achieved was retained for analysis. Subsequently, the submaximal endurance task was performed. Participants were required to isometrically sustain the weight of their trunk with an external load of 10 pounds while crossing their arms over the chest to hold the weight, as long as possible. The test concluded when the subject could no longer maintain the reference position for more than three seconds [29]. Any deviation from the reference target for more than three seconds was considered as task failure. The participant’s position was monitored by an evaluator. Participants were informed when they began to deviate, allowing them the opportunity to correct their posture. If they were unable to correct it within the three-second window, the test was terminated. The duration of the test was recorded, and surface EMG was collected using two bipolar electrodes (one on each side of the trunk) (Model DE2.1, Delsys Inc., Boston, MA, USA) placed on the erector spinae (ES) muscles (L3 level). The skin was shaved and cleaned with fine sandpaper (Red DotTrace Prep; 3M, St. Paul, MN) and friction alcohol (70% ethyl alcohol). The EMG electrodes were positioned by the same evaluator, and the placement was marked with a pencil to ensure fidelity between evaluation sessions. During the data collection, EMG signals were sampled at 1000 Hz and converted to digital (12-bit A/D converter; Input range ±5V; 256 channels EMG-USB2; OTBioelettronica). Signals were linearly amplified by a factor between X1000 and X10000 to account for variability in participants’ skin impedance. This variable amplification ensures that the signal fully use the dynamic range of the acquisition card improving signal resolution. The amplified signals were then analyzed using MATLAB (v.2023b; The MathWorks, Natick, MA). Signals obtained from the two EMGs underwent digital band-pass filtering using a 4^th^-order Butterworth filter with a frequency range of 20 to 450 Hz. Notch filters, implemented as 2^nd^ order Butterworth filters, were also applied to eliminate interference from the 60 Hz power line and its harmonics. To evaluate the quality of the EMG signals, amplitude spectra were computed using a Fast Fourier transform and were visually screened. EMG measurements, specifically the median frequency (MF) and the slope of the MF, were calculated. The MF was calculated for both sides using overlapping moving windows of 0.5 seconds. The slope of the MF was determined from all MF values obtained during the endurance test for both sides. Subsequently, the MF values were averaged over the entire duration of the test. The MF and slope values from both the left and right ES sides were then averaged since there was no statistically significant difference (p>0.05) between the two sides. This approach aimed to enhance the robustness of the comparison between times of day and reduce variability. These fatigue measurements of the back muscle during the Sorenson test have good intersession reliability [30].

### Disc height evaluation

**Fig 1:**
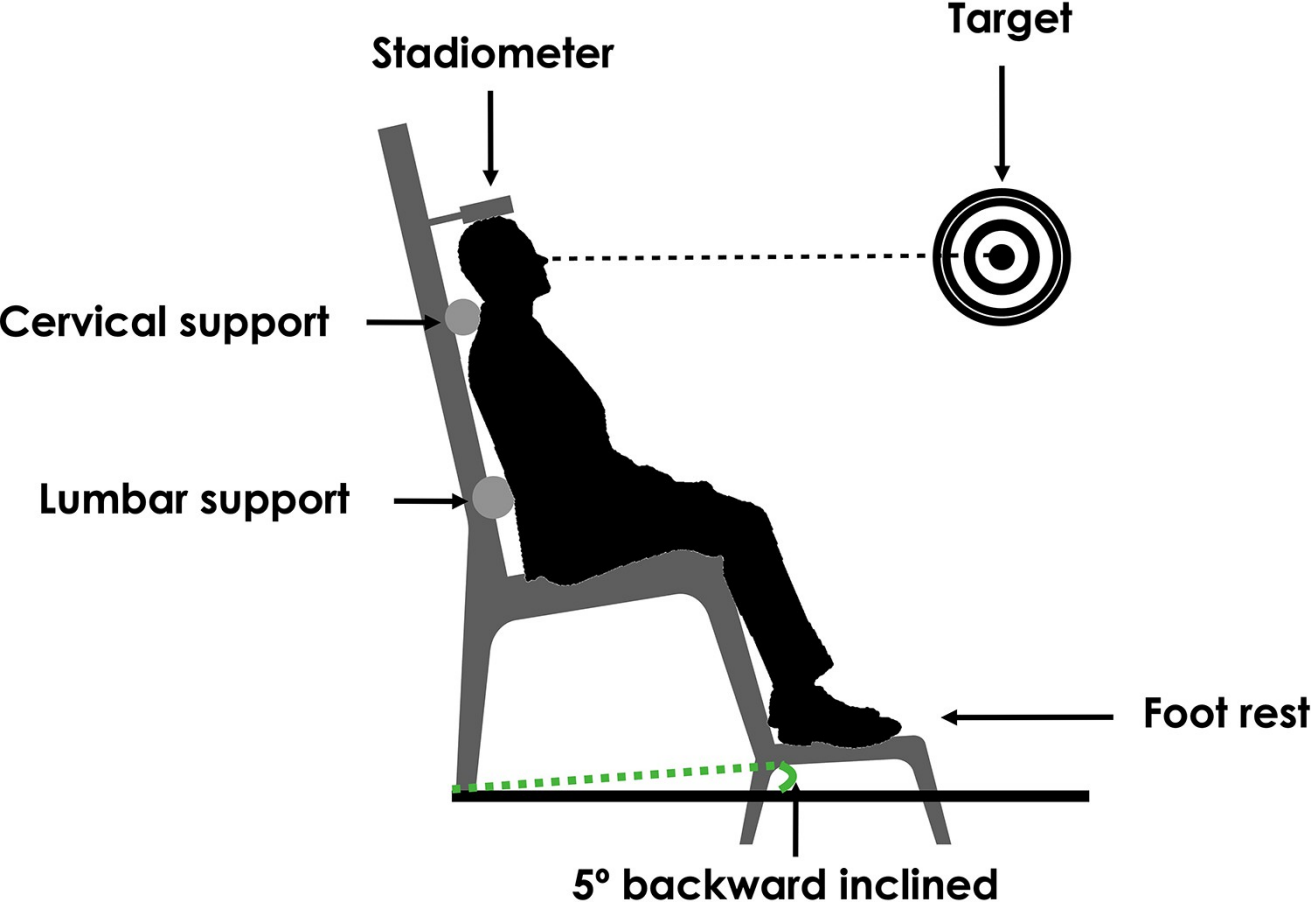
Disc height evaluation with stadiometer.

The stadiometer (235 HeightronicTM Digital Stadiometer, Measurement Concepts, Quick Medical, Snoqualmie, WA. Precision: 0.01 mm) consists of a digital measuring rod mounted on a wooden frame. Changes in sitting height measured using this stadiometry setup are strongly correlated with lumbar spine disc height changes measured using ultrasonography, as shown in a prior study [31]. Individually tailored cervical and lumbar supports are placed behind the respective segments of the participant and is secured to the wooden frame in order to maintain the physiological curvatures of the spine, thus avoiding variations in height due to modifications in the concavities/convexities of the curvatures. Fig 1 illustrates the participant’s position during the evaluation of discs height. Stadiometric measurements were conducted three times by the same investigator, and the average of these three measurements was used for the analysis. Stadiometric measurements have shown very good reliability, repeatability and validity [31–33].

### Postural stability

Postural stability was assessed using a force platform (Bertec Corporation®, Columbus, OH, USA). Participants were required to stand on the platform for continuous periods of 30 seconds for a total of three trials per condition. The three postural conditions were as follows: Bipedal standing position with eyes open, bipedal standing position with eyes closed, and bipedal standing position with eyes open while standing on an unstable surface (10 cm thick foam). For all three conditions, arms were maintained at each side of the body and the distance between the two feet corresponded to shoulder width. These conditions were chosen to disrupt one of the sensory systems (eyes close; vision and unstable surface; somatosensory information), necessitating a greater contribution from other sensory systems [34]. Trials during which there was a loss of balance or foot movement were excluded, and an additional trial was conducted. In both eyes opened conditions, participants focused on a target three meters away at eye level. Participants were asked to stand as still as possible. Data collection began once participants felt ready and stable. The order of conditions was randomized across participants. The foot position of each participant was traced on a paper sheet covering the force platform, ensuring consistent placements for each trial and time of day. This protocol is following the recommendation of a systematic review for maximizing the reliability of center of pressure (COP) data [35]. Kinetic data were acquired using a force plate with a sampling frequency of 100 Hz, and subsequent low-pass filtering at 10 Hz was applied through a 2^nd^ order Butterworth filter using Matlab. The displacement of the COP in both the anteroposterior (AP) and mediolateral (ML) directions, referred to as COP_AP_ and COP_ML_, respectively, was determined using the following equations:

$$COP_{AP}=M_{ML}/F_{z}$$

$$COP_{ML}=M_{AP}/F_{z}$$

where F_z_ represents the vertical ground reaction force applied to the participant’s center of gravity, and M_AP_​ and M_ML_​ are the moments of force exerted on the surface of the force plate along the AP and ML directions, respectively. Balance parameters, including the 95% confidence ellipse area of COP (in mm^2^) [36], COP velocity (in mm/s), and root mean square (RMS) (in mm) for both COP_AP_ and COP_ML_. To assess the magnitude of fluctuations normalized by the mean, the mean COP values along the AP and ML directions were subtracted from the COP data before computing RMS. COP velocity was determined by dividing the sway length by the recording duration (30 seconds) in both ML and AP axes. These parameters were calculated over the entire duration of the 30-second trials and the average of the three trials was used for analysis. These COP measurements, extensively used for evaluating postural stability, were previously detailed in our earlier work [37].

### Daily physical activity assessment

Between the two laboratory measurements, during the workday, participants wore an activity monitor (activPAL; PAL Technologies Ltd., Glasgow, UK) for objective data collection regarding daily physical activity levels, which was used to assess workload. The activPAL is a validated tool that provides accurate individual estimates of active and sedentary behaviors in healthy adults [38]. The monitor was worn continuously on the non-dominant lower limb, and collected the number of steps taken, time (in hours) spent in sedentary (sitting) position, time spent standing, the number of transitions from sitting to standing and metabolic equivalent of tasks (METs).

### Statistical analysis

The statistical analysis was conducted with SPSS Statistics for Mac, version 28 (SPSS Inc., IBM Corp., Armonk, NY, USA). Descriptive statistics were used to characterize participants sociodemographic information’s and the characteristics of their work. The Shapiro-Wilk test was conducted for all continuous variables to assess data distribution. Participants’ characteristics (i.e., age, gender, years of seniority weight, height, and job type) were summarized using frequency distributions for categorical variables and means and standard deviations for continuous variables. For group comparisons, the independent samples t-test was used for continuous variables, and the chi-square test for categorical variables. Comparisons between the older and younger worker groups for muscle fatigue and disc height variables were conducted, using repeated measures analysis of variance (ANOVA) with two measurement times (2X2) (before and after the workday (time of day)) across two independent groups (older and younger workers (age)). Postural stability was analyzed using a three-way repeated measures ANOVA (2X2X3) for Age, Time of day, and Posture, following the approach of a previous study [39]. Participants’ characteristics were used as controlled variables in the analyses. Specifically, when the two groups exhibited significant differences (p ≤ 0.05), these variables were integrated into an analysis of covariances (ANCOVA) model. This model allowed the calculation of age effects while controlling for the influence of other potentially confounding variables that could impact the relationship between age and the measured physical and physiological changes before and after the workday. Bonferroni post hoc tests were used when necessary. Effect sizes were reported using partial eta-squared (η^2^), classified as small (0.01), medium (0.06), and large (≥ 0.14) [40]. Results are presented using the mean and standard deviation. A significance level of p ≤ 0.05 was used for all statistical assessments.

## Results

### Participant characteristics

A total of 40 participants, with 20 in Group 1 (young workers: ≤50 years) and 20 in Group 2 (older workers: >50 years), were recruited. Socio-demographic characteristics and job satisfaction outcomes for each group are presented in Table 1. The two groups did not differ significantly in any socio-demographic variable, except for mean age, years of seniority, and average weight (kg). Older participants had a significantly higher number of years of seniority, totaling 13.04 ± 13.56 years, compared to 2.49 ± 2.65 years for younger workers (p = 0.003). The average weight of older workers (79.13 ± 16.30 kg) was also significantly higher (p = 0.033) when compared to younger workers (70.27 ± 13.04 kg). Participants’ weight was incorporated into an analysis of covariance (ANCOVA) model due to differences between groups. This allowed for the control of possible confounding effect of weight on strength, endurance, and muscle fatigue [41], as well as on disc height [42] and postural stability [43].

**Table 1:** Sociodemographic characteristics.

Characteristics	Group 1 (N = 20)	Group 2 (N = 20)
n (%) or mean ± SD		
Age (years)*	29.89 ± 7.23	59.40 ± 5.29
**Gender**	M : 10 (50.0) F : 10 (50.0)	M : 12 (60.0) F : 8 (40.0)
Years of seniority *	2.49 ± 2.65	13.04 ± 13.56
Weight (kg)*	70.27 ± 13.04	79.13 ± 16.30
**Height (cm)**	169.98 ± 7.91	170.50 ± 10.70
**Job type**		
**Teaching**	4 (20.0)	4 (20.0)
**Administrative work**	5 (23.8)	4 (20.0)
**Labor workers**	9 (42.9)	7 (35.0)
**Other**	2 (9.5)	5 (25.0)
**Job satisfaction**		
**General**	83.25 ± 11.91	81.61 ± 11.93
**Intrinsic**	51.38 ± 6.76	50.72 ± 7.75
**Extrinsic**	24.19 ± 3.83	23.11 ± 5.86

Significant differences (p<0.05) are denoted by asterisks (*); F: Female; M: Male; kg: kilograms; cm: centimeters; SD: Standard deviation.

For the entire sample, job satisfaction data from seven participants could not be collected as their self-reported questionnaires could not be retrieved at the end of the workday or were not returned via email, even after multiple reminders. Nevertheless, no statistically significant difference in overall (p = 0.692), intrinsic (p = 0.796), and extrinsic job satisfaction (p = 0.536) was observed between younger and older workers. According to the 34 completed questionnaires, nine participants reported a low level of job satisfaction, while 25 participants showed a high level of job satisfaction.

### Muscle fatigue

#### Maximum muscle force

**Fig 2:**
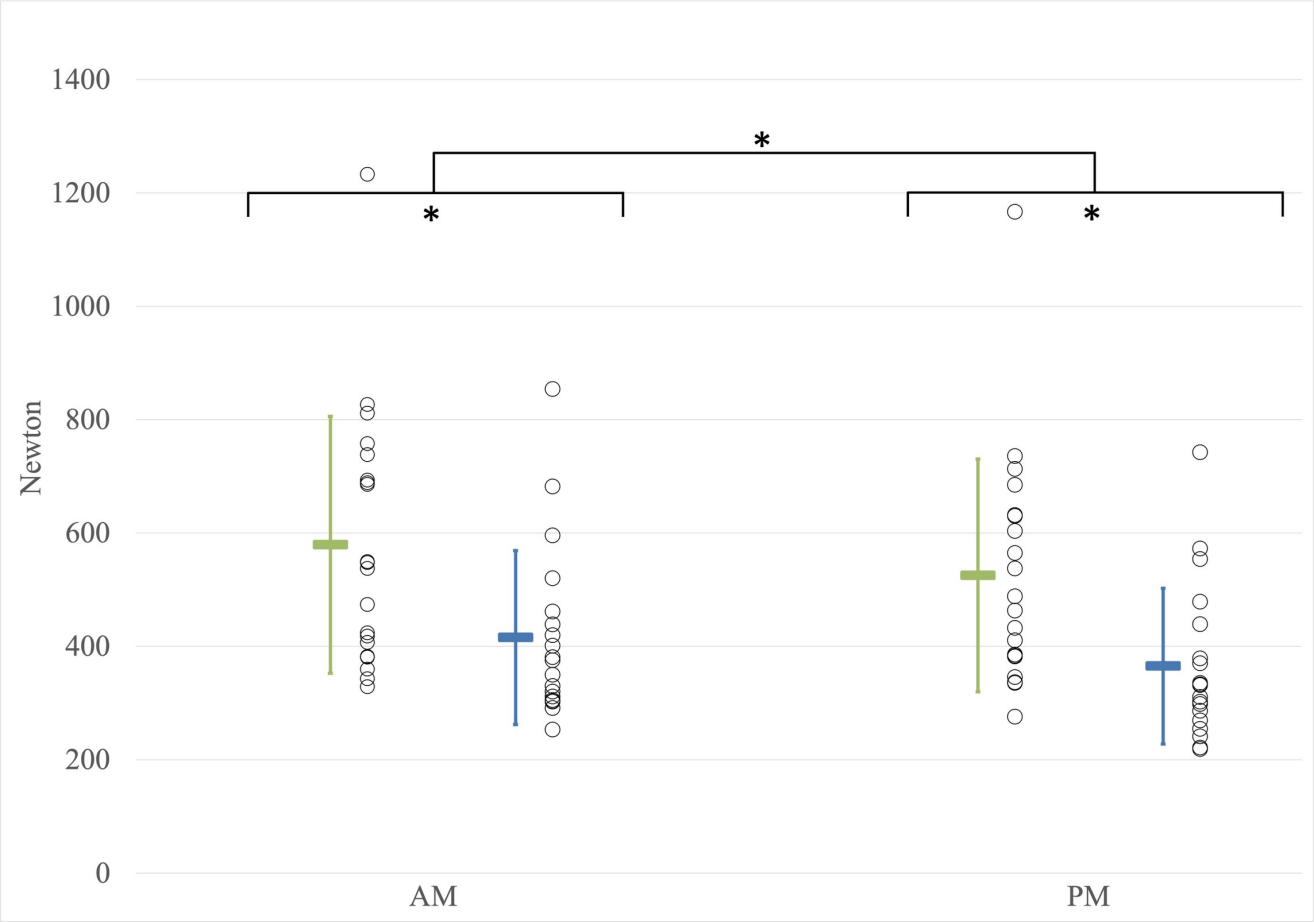
Maximum muscle force. AM: Before the workday; kg: kilograms; PM: After the workday; Younger workers; Older workers. Asterisks (*) indicate significant differences (p < 0.05). Individual data points are represented by circles, while the mean values are depicted as colored rectangles. Error bars represent the standard deviation.

Data related to the maximal muscle force were collected for 39 out of 40 workers as aberrant data were removed (20 young workers; 19 older workers). A significant effect of the time of day on MVIC was observed (F(1,37) = 24.649; p<0.001; η^2^ = 0.400) (Fig 2). Workers exhibit higher maximum force in the morning compared to the end of the workday. A main group effect was observed on maximum force (F(1,37) = 7.688; p = 0.009; η^2^ = 0.172) with younger workers exhibiting higher force compared to older workers. No significant interaction between the time of day and group was observed (F(1,37) = 0.028; p = 0.867; η^2^<0.001). The addition of weight as a covariate (ANCOVA) revealed no significant effect of the time of day on maximum force (F(1,36) = 3.141; p = 0.085; η^2^ = 0.080).

#### Time to task failure in the modified sorensen test

**Fig 3:**
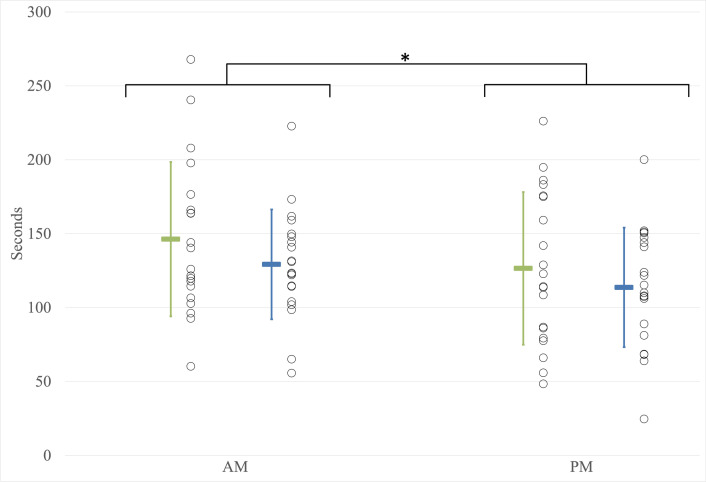
Duration of maintaining posture in the modified sorensen test. AM: Before the workday; PM: After the workday; Younger workers; Older workers. Asterisks (*) indicate significant differences (p < 0.05). Individual data points are represented by circles, while the mean values are depicted as colored rectangles. Error bars represent the standard deviation.

A statistically significant effect of the time of day on the time to task failure during the modified Sorensen test (F(1,38) = 28.937; p<0.001; partial η^2^ = 0.432) was observed. Workers exhibited longer lumbar extension time to task failure in the morning compared to the end of the day (Fig 3). No significant group effect on the time to task failure during the modified Sorensen test (F(1,38) = 1.123; p = 0.296; partial η^2^ = 0.029) was observed. No significant interaction between the time of day and group was observed (F(1,38) = 0.419; p = 0.521; partial η^2^ = 0.011). Adding weight as a covariate in the ANCOVA revealed no significant effect of the time of day on the time to task failure during the modified Sorensen test (F(1,37) = 0.660, p = 0.422, partial η^2^ = 0.013).

#### Electromyographic measurements

**Fig 4:**
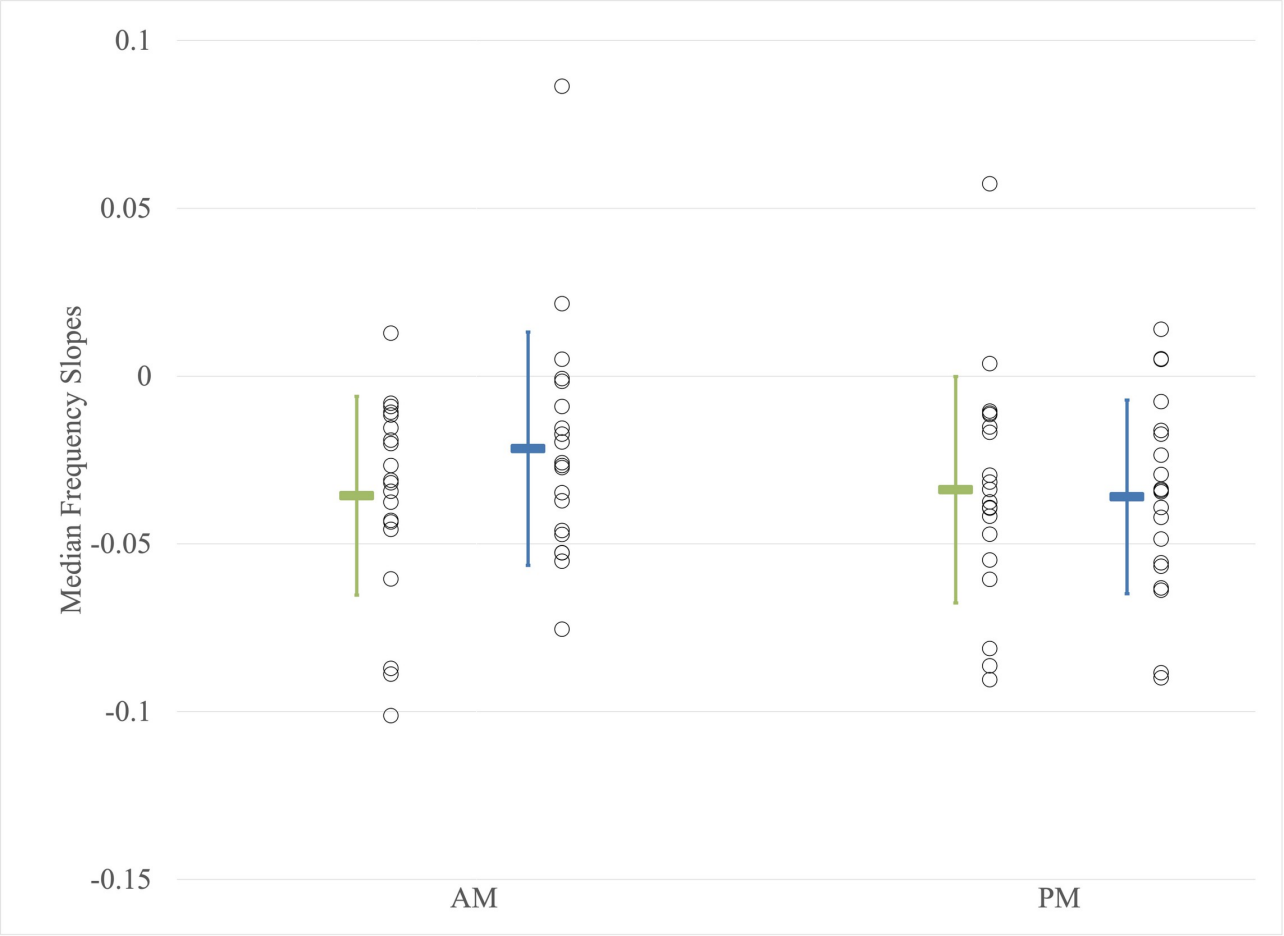
Measures of median frequency slope. AM: Before the workday; PM: After the workday; Younger workers; Older workers. Individual data points are represented by circles, while the mean values are depicted as colored rectangles. Error bars represent the standard deviation.

Fig 4 illustrates the comparison between the time of day and the mean slopes of the MF. No significant effect of the time of day on the MF slope was observed (F(1,38) = 1.209; p = 0.278; η^2^ = 0.031). No significant main group effect was noted (F(1,38) = 0.519; p = 0.476; η^2^ = 0.013). Additionally, no significant interaction between the time of day and group was observed (F(1,38) = 1.983; p = 0.167; η^2^ = 0.050). The same results were found with the addition of weight as a covariate (ANCOVA).

**Fig 5:**
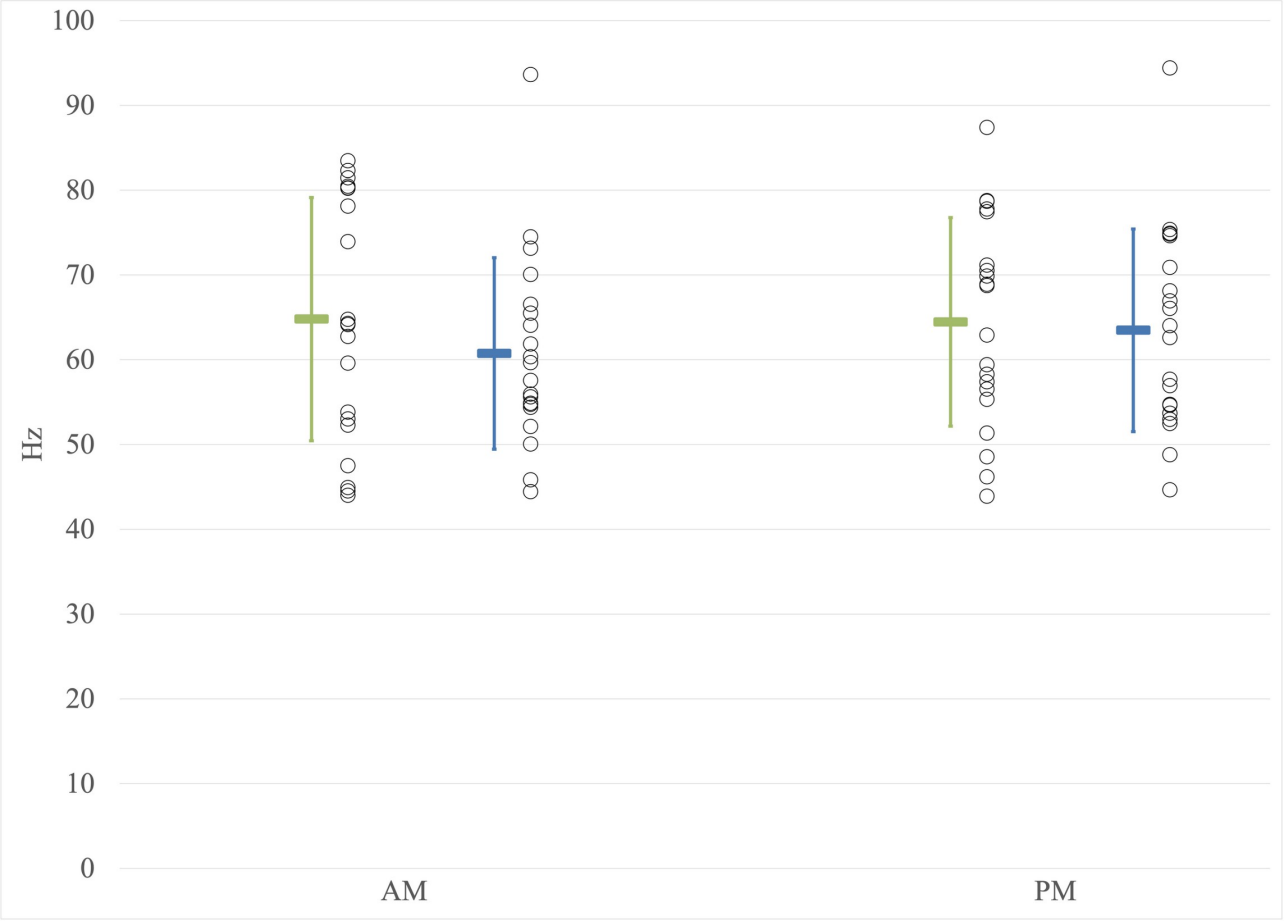
Measures of median frequencies. AM: Before the workday; PM: After the workday; Hz: Hertz; Younger workers; Older workers. Individual data points are represented by circles, while the mean values are depicted as colored rectangles. Error bars represent the standard deviation.

For the mean values of the MF (Fig 5), no significant effect of the time of day on the MF value was observed (F(1,38) = 2.062; p = 0.159; η^2^ = 0.051). No significant main effect of group was observed (F(1,38) = 0.424; p = 0.519; η^2^ = 0.011). Additionally, no significant interaction between the time of day and group was identified (F(1,38) = 3.392; p = 0.073, η^2^ = 0.082). The same results were found with the addition of weight as a covariate (ANCOVA).

### Disc height variations

**Fig 6:**
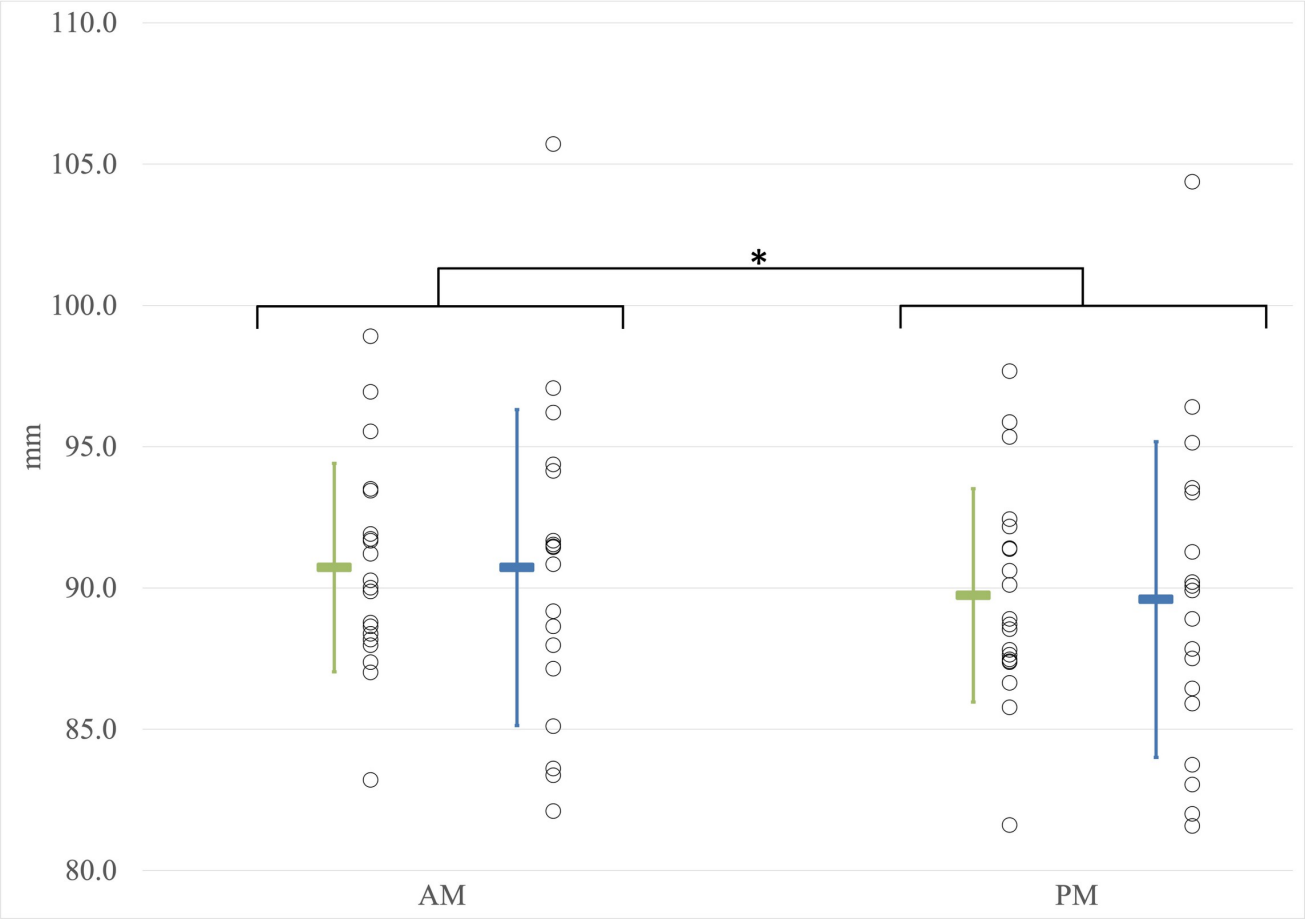
Measurements of disc height. AM: Before the workday; mm: millimeters; PM: After the workday; Younger workers; Older workers. Asterisks (*) indicate significant differences (p < 0.05). Individual data points are represented by circles, while the mean values are depicted as colored rectangles. Error bars represent the standard deviation.

Data related to the variation in disc height were collected for 39 out of 40 workers as aberrant data were removed (20 young workers; 19 older workers). A significant effect of the time of day on disc height measurement by stadiometry was observed (F(1,37) = 169.091; p<0.001; η^2^ = 0.820). Workers had a greater disc height in the morning compared to the end of the workday (Fig 6). No significant main effect of group was identified (F(1,37) = 0.002; p = 0.963; η^2^<0.001). There was no significant interaction between the time of day and group (F(1,37) = 0.849; p = 0.363; η^2^ = 0.022). The addition of weight as a covariate (ANCOVA) revealed no significant effect of the time of day on disc height measurement (F(1,35) = 1.189; p = 0.283; η^2^ = 0.032).

### Postural stability

The results of the statistical analyses (ANOVAs and ANCOVAs) on postural stability variables are detailed in the Supplementary material. Postural stability data were collected for 36 out of 40 workers as aberrant data were removed (18 young workers; 18 older workers). All ANOVA results are presented in the supporting information section (S1 Table).

### Effect of postural conditions

The ANOVA showed a significant effect of stability conditions on all examined COP variables. Significant effects were observed specifically for COP surface area (F(2,68) = 93.557; p<0.001; η^2^ = 0.733), COP velocity in AP (F(2,68) = 81.721; p<0.001; η^2^ = 0.706) and ML (F(2,68) = 141.930; p<0.001; η^2^ = 0.807), COP RMS in AP (F(2,68) = 89.224; p<0.001, η^2^ = 0.724) and ML (F(2,68) = 207.011; p<0.001; η^2^ = 0.859). Post hoc tests revealed that for all COP variables, more challenging postural stability conditions led to significantly higher values compared to conditions on a stable surface (eyes open and eyes closed) (all with p<0.001). The condition with eyes closed had significantly higher values for ML velocity, as well as AP velocity and RMS, compared to the stable condition with eyes open (respectively: p<0.001, p<0.001, p = 0.003). The same results were found with the addition of weight as a covariate (ANCOVA).

### Main effect of group

The ANOVA revealed a significant effect of group on velocity (F(1,34) = 14.642; p<0.001, η^2^ = 0.301) and RMS (F(1,34) = 5.877; p = 0.021; η^2^ = 0.147) in AP. However, when weight was included as a covariate (ANCOVA), a significant effect of group on AP velocity was still present (F(1,33) = 14.868, p = 0.001; η^2^ = 0.311), while age no longer had a significant effect on RMS in AP (F(1,33) = 3.608; p = 0.066; η^2^ = 0.099). Moreover, the results of the ANCOVA revealed a significant effect of group on ML velocity, which was not initially observed in the ANOVA (F (1,33) = 6.017; p = 0.020, η^2^ = 0.154).

### Main effect of time of day

The ANOVA revealed a significant effect of the time of day on ML COP velocity (F(1,34) = 13.234; p<0.001; η^2^ partial = 0.453). ML COP velocity in the morning was significantly higher than that measured after the workday. However, this effect was no longer present when weight was included in the ANCOVA (F(1,33) = 2,400; p = 0.131; η^2^ = 0.068).

### Interactions

The ANOVA revealed a significant interaction between postural conditions (eyes open, eyes closed, and unstable surface) and age on AP COP velocity (F(2,68) = 3.234; p = 0.046; η^2^ partial = 0.087). Post hoc tests showed that for all postural conditions, older workers had higher velocity values than younger workers (p<0.001). Additionally, both younger and older workers exhibited higher velocity values in unstable conditions compared to the eyes closed condition (young workers: p = 0.003; older workers: p = 0.006) and the eyes open condition (young workers: p<0.001; older workers: p<0.001). Both younger and older workers also showed higher velocity values for the eyes closed condition compared to the eyes open condition (young workers: p<0.001; older workers: p<0.001). Furthermore, when weight was added as a covariate, the ANCOVA showed the same interaction, along with consistent post hoc results.

### Daily physical activity level

Table 2 presents the results related to the daily physical activity level obtained using the activity monitor. Two participants (one in Group 1 and one in Group 2) were removed due to aberrant data. No significant differences were observed between the group of young workers and the group of older workers regarding daily step count (t(36) = 1.175; p = 0.248), energy expenditure (t(36) = 0.672; p = 0.506), time spent sitting (t(36) = -1.935; p = 0.061) and standing (t(36) = 1.366; p = 0.180), and the number of transitions from sitting to standing (t(36) = -0.477; p = 0.636).

**Table 2:** Daily physical activity levels.

Variables	Group 1 (N = 19)	Group 2 (N = 19)
mean ± SD		
**Number of steps**	5481.21 ± 1531.43	4846.37 ± 1788.36
**MET**	12.59 ± 1.06	12.36 ± 1.01
**Time spent standing (h)**	4.89 ± 1.25	4.29 ±1.45
**Time spent sitting (h)**	2.97 ± 1.08	3.75 ±1.38
**Number of transitions from sitting to standing**	24.11 ± 13.21	26.16 ± 13.31

h: hours; MET: Metabolic Equivalent of Task; SD: Standard deviation

## Discussion

This study aimed to assess the impact of physical efforts made during a workday on endurance, muscle fatigue of trunk muscles, disc height variations in the lumbar spine, and postural stability in older workers, while comparing the effects of a similar workday on younger workers. The initial hypothesis was that, for a similar workload, older workers would experience more significant physiological and biomechanical changes. However, our findings did not support this hypothesis, as no significant interaction between age and time of day was observed. This conclusion was reached despite both groups having similar sociodemographic characteristics, with the only notable difference being in participants’ weight, with older workers having significantly higher weight. The study found that for both groups, the workday had a significant effect on physiological variables for several parameters: decreased endurance and maximal force generating capacity of the ES muscles, decreased disc height, and a decrease in the COP lateral displacement velocity. Additionally, the study identified age-related effects on maximal force production and several variables of postural stability. However, incorporating weight as a covariate in the statistical analyses revealed that these differences could be partially explained by the weight discrepancy between older and younger workers.

These findings partly align with those of other studies, which suggest that age, specifically the process of healthy aging, does not seem to consistently affect muscle fatigue. Indeed, studies by Christie et al. (2011) and Boocock et al. (2020) demonstrated that despite greater muscle activity during a task, older individuals tended to experience less muscle fatigue during isometric muscle contractions [8, 20]. Moreover, Tsuboi et al. (2013) aimed to quantify differences in the muscular activity of lumbar ES muscles between young and older participants, using various objective indicators of muscle fatigue during the Sorensen endurance test [44]. Although no significant difference in performance (time in seconds) during the endurance test was identified between young and older participants, a difference in the MF slopes and in the average frequency of the power spectrum was noted. These were significantly lower in older male participants compared to young male participants, suggesting greater proportion of type 1 fibers, and indicating less accumulation of muscle fatigue in older men. However, no significant difference was identified between young and older female participants [44]. Additionally, one study by Champagne (2009) found no significant difference between older and younger individuals on lumbar extensors muscle endurance, MVIC values and percentage reduction in MVIC after the endurance test using the modified Sorensen test [27]. In the current study, the lack of consistent difference between age groups on muscle fatigue could be explained by the fact that our study included only active workers. Older individuals who maintain a consistently high level of physical activity through their work may experience less decline in muscle strength and endurance compared to their sedentary counterparts. This can be explained by the well-known effect of regular exercise on preserving muscle strength and function with age [45, 46]. Further investigations are warranted in sedentary workers to comprehensively understand the relationship between physical activity levels, aging, and muscle health.

The age-related effects on postural stability are well known. A systematic review and meta-analysis of 38 international studies revealed a statistically significant increase in AP and ML COP displacement in the "eyes closed" condition compared to an "eye open" conditions among older individuals, while younger individuals showed similar values for both conditions, indicating better adaptation to visual deprivation [47]. Furthermore, differences in COP displacement and velocity were identified between the two age groups, with older people exhibiting 20 to 50% higher COP displacement and velocity than young participants [47]. Additionally, a study by Duarte (2022) showed differences in postural strategies deployed in anticipation of a perturbation between younger and older participants [48]. These were characterized by a longer latency in initiating muscle activity in the lower limb and lumbar spine muscles, as well as a higher initiation time of COP displacement among older participants [48]. Our findings align with previous studies that have demonstrated higher COP velocity in older adults. To our knowledge, there is no study investigating these effects combined with physical efforts made during a workday. Overall, our study shows that there was no age difference on the impact of a workday on postural stability. Although age differences were found and could be attributed to participants’ weight, both groups exhibited similar adaptations in both AM and PM measurements. These similarities indicate that both groups adapt similarly to the workload throughout the workday.

In our investigation, no discernible age-related difference in disc height was observed, instead, time of day difference was found. Nevertheless, this difference appears to be partially attributable to body weight. The variations in IVD height are postulated to arise from the redistribution of water content within the IVD [49]. This water redistribution is contingent upon the applied load on the disc. Specifically, individuals with higher BMI may passively exert increased loading on the intervertebral discs throughout the day, resulting in a more pronounced reduction in water content within the IVD and thus explained the reduce IVD height. Supporting this hypothesis, Urquhart et al. (2014) found that, in community-based individuals, obesity was associated with reduced total and mean disc heights in the lumbar spine [42].

### Perspectives

The data from this study suggests that age may not be the primary determinant of the impact of a workday on older workers but could be their physical condition that plays a determining role (indirectly measured by workers’ weight). Indeed, most of the differences observed between older and younger workers in initial variance analyses disappeared in the analyses of covariances that incorporated workers’ weight. The physical requirements for work can vary greatly depending on the job type. Indeed, many professions demand specific physical abilities from workers. In this context, preserving the physical capacities of older workers is an important factor in maintaining occupational health, work productivity, and ultimately promoting the longevity of experienced workers [50, 51]. These findings suggest that measures implemented to optimize the work activity while reducing the overall physical strain associated with the tasks job do not necessarily need to target age categories but could rather focus on the physical abilities of the workers and the requirements of the work environment. Future studies should pay particular attention to job characteristics, physical activity levels required, and psychosocial and environmental factors specific to the workplace. The UK Health and Safety Executive also concludes that employers should not assume that certain jobs are too demanding for older workers, as many jobs are supported by technology, which can absorb the physical strain. Consulting and involving older workers when considering which control measures to put in place should be promoted to manage health and safety in the work environment [52]. Despite evidence suggesting that older workers are less prone to workplace injuries due to their experience and precautionary measures [53], age-related prejudices and beliefs persist in work environments [54, 55]. Managers, while more inclined to hire older workers because of the labor shortages, often uphold practices that may limit their effectiveness, particularly in task assignment [56]. While reducing workload may be beneficial for some older workers, the heterogeneous nature of aging necessitates consideration of individual differences in adapting to work-related challenges.

### Strengths and limitations

In general, studies conducted with a limited number of workers have some limitations that need to be considered when interpreting the results. Firstly, the exploratory nature of this study and its small sample size limits the generalizability of the data. In this study, the recruited workers and the results obtained, especially regarding the physical efforts deployed during the workday, restrict the generalization to other groups of workers who may work in industries or work environments where the efforts exerted, and their impacts are different. It is also possible that the workers who participated in the study may not be representative of their colleagues who declined to participate. For example, healthier workers may have been more willing to participate in the project, which included tasks requiring sustained or repeated physical efforts. In total, 30 different workplaces were contacted, and despite a relatively satisfactory level of interest shown (40% of workplaces interested in the project, totaling nearly 174 employees), less than a quarter of the employees approached agreed to participate in the project. Additionally, although workers in both groups have similar average characteristics (except for weight), it is possible that the heterogeneity in job types, environments, and tasks may have limited our ability to identify significant differences between younger and older workers.

Another limitation of this study is the calculation of the CoP for the unstable condition. Although incorporating foam thickness into the CoP calculation typically enhances accuracy, the variability of the foam compression under participants’ weight was not accounted for. As participants stood on the foam, initially 10 cm in height, its low density caused it to compress to approximately 1 cm, with heterogeneous pressure distribution influenced by participants’ weight, further complicating the precision of thickness estimation. Consequently, no adjustments were made to the CoP calculation to avoid introducing errors into the results.

Finally, other compensatory strategies that participants may have used to complete the modified Sorensen task, such as recruiting the hamstrings, gluteus maximus, or other regions of the erector spinae, were not assessed. Evaluating these strategies could have provided additional explanations for the results, such as redistribution of muscle fatigue across muscles or muscle regions. Future research should measure the entire back extensor muscle group to gain a comprehensive understanding of muscle fatigue adaptations.

## Conclusion

In conclusion, this study aimed to examine the impact of physical efforts during a workday on various physiological and biomechanical parameters in older and younger workers. Contrary to the initial hypothesis, no significant interaction between age and time of day was observed, indicating that, for a similar workload, both age groups experienced similar changes. Despite age-related effects on maximal force production and postural stability, incorporating weight as a covariate revealed that these differences were partially explained by the weight discrepancy between older and younger workers. These findings suggest that, in active older individuals, maintaining a consistently high level of physical activity through work may mitigate the decline in muscle strength and endurance typically associated with aging. The study suggests that physical fitness should be considered as a factor in understanding age-related changes in muscle health and biomechanics. Future investigations of sedentary workers are warranted to comprehensively explore the relationship between physical activity levels, aging, and muscle health.

## Supporting information

S1 Table Statistical analysis for postural stability variables. AM: Morning; PM: Afternoon; COP: Center of Pressure; Hz: Hertz; mm: millimeters; RMS: Root Mean Square; EC: Eyes Closed; EO: Eyes Open; Statistically significant result are presented in bold.
(DOCX)
