# Supplementary material for: The influence of workload on muscle fatigue, tissue properties, and postural stability in older and younger workers
Source: PLoS One. 2025 Jan 3;20(1):e0316678. doi: 10.1371/journal.pone.0316678 (PMC11698325; doi:10.1371/journal.pone.0316678)
Supplement: S1 Table — AM: Morning; PM: Afternoon; COP: Center of Pressure; Hz: Hertz; mm: millimeters; RMS: Root Mean Square; EC: Eyes Closed; EO: Eyes Open; Statistically significant result are presented in bold. (DOCX) [file pone.0316678.s001.docx]

| Variables | Posture | Time of day | Groups | Mean (SD) | Statistics | | | | | | | | | | | | | |
| --- | --- | --- | --- | --- | --- | --- | --- | --- | --- | --- | --- | --- | --- | --- | --- | --- | --- | --- |
|  |  |  |  |  | Posture | | Age | | Time of day | | Posture*age | | Time of day*  Age | | Posture*  Time of day | | Posture*  Time of day*Age | |
|  |  |  |  |  | ANOVA | ANCOVA | ANOVA | ANCOVA | ANOVA | ANCOVA | ANOVA | ANCOVA | ANOVA | ANCOVA | ANOVA | ANCOVA | ANOVA | ANCOVA |
|  |  |  |  |  | df (2.68) | df (2.66) | df (1.34) | df (1.33) | df (1.34) | df (1.33) | df (2.68) | df (2.66) | df (1.34) | df (1.33) | df (2.68) | df (2.66) | df (2.68) | df (2.66) |
| COP area (mm^2^) | EO | *AM* | Young | 91.85 (48.95) | **F=93.557 P<0.001 η²=0.733** | **F=8.286 P=0.006 η²=0.201** | F=2.532 P=0.121 η²=0.069 | F=2.120 P=0.155 η²=0.060 | F=1.199 P=0.281 η²=0.034 | F=0.637 P=0.431 η²=0.019 | F=1.055 P=0.317 η²=0.030 | F=1.755 P=0.194 η²=0.050 | F=0.015 P=0.904 η²=0.000 | F=0.093 P=0.762 η²=0.003 | F=0.563 P=0.472 η²=0.016 | F=0.458 P=0.519 η²=0.014 | F=0.171 P=0.703 η²=0.005 | F=0.298 P=0.608 η²=0.009 |
|  |  |  | Old | 130.26 (88.36) |  |  |  |  |  |  |  |  |  |  |  |  |  |  |
|  |  | *PM* | Young | 101.71 (63.27) |  |  |  |  |  |  |  |  |  |  |  |  |  |  |
|  |  |  | Old | 140.96 (93.39) |  |  |  |  |  |  |  |  |  |  |  |  |  |  |
|  | EC | *AM* | Young | 121.34 (72.90) |  |  |  |  |  |  |  |  |  |  |  |  |  |  |
|  |  |  | Old | 183.64 (170.28) |  |  |  |  |  |  |  |  |  |  |  |  |  |  |
|  |  | *PM* | Young | 125.92 (93.39) |  |  |  |  |  |  |  |  |  |  |  |  |  |  |
|  |  |  | Old | 165.27 (109.39) |  |  |  |  |  |  |  |  |  |  |  |  |  |  |
|  | Unstable | *AM* | Young | 448.16 (180.78) |  |  |  |  |  |  |  |  |  |  |  |  |  |  |
|  |  |  | Old | 559.79 (249.04) |  |  |  |  |  |  |  |  |  |  |  |  |  |  |
|  |  | *PM* | Young | 474.58 (274.06) |  |  |  |  |  |  |  |  |  |  |  |  |  |  |
|  |  |  | Old | 549.55 (396.25) |  |  |  |  |  |  |  |  |  |  |  |  |  |  |
| Velocity (COP _AP_) (mm/s) | EO | *AM* | Young | 5.89 (1.21) | **F=81.721 P<0.001 η²=0.706** | **F=10.185 P<0.001 η²=0.236** | **F=14.642 P<0.001 η²=0.301** | **F=14.037 P<0.001 η²=0.298** | F=2.890 P=0.098 η²=0.078 | F=1.680 P=0.204 η²=0.048 | **F=3.234 P=0.046 η²=0.087** | **F=4.500 P=0.018 η²=0.120** | F=0.972 P=0.331 η²=0.028 | F=0.388 P=0.538 η²=0.012 | F=1.653 P=0.206 η²=0.046 | F=1.113 P=0.327 η²=0.033 | F=0.009 P=0.974 η²=0.000 | F=0.091 P=0.913 η²=0.003 |
|  |  |  | Old | 7.39 (2.07) |  |  |  |  |  |  |  |  |  |  |  |  |  |  |
|  |  | *PM* | Young | 5.71 (0.87) |  |  |  |  |  |  |  |  |  |  |  |  |  |  |
|  |  |  | Old | 7.65 (1.96) |  |  |  |  |  |  |  |  |  |  |  |  |  |  |
|  | EC | *AM* | Young | 8.62 (1.26) |  |  |  |  |  |  |  |  |  |  |  |  |  |  |
|  |  |  | Old | 12.39 (4.88) |  |  |  |  |  |  |  |  |  |  |  |  |  |  |
|  |  | *PM* | Young | 7.77 (1.49) |  |  |  |  |  |  |  |  |  |  |  |  |  |  |
|  |  |  | Old | 11.90 (4.53) |  |  |  |  |  |  |  |  |  |  |  |  |  |  |
|  | Unstable | *AM* | Young | 11.23 (1.65) |  |  |  |  |  |  |  |  |  |  |  |  |  |  |
|  |  |  | Old | 14.79 (5.28) |  |  |  |  |  |  |  |  |  |  |  |  |  |  |
|  |  | *PM* | Young | 10.68 (2.00) |  |  |  |  |  |  |  |  |  |  |  |  |  |  |
|  |  |  | Old | 14.59 (4.77) |  |  |  |  |  |  |  |  |  |  |  |  |  |  |
| Velocity (COP _ML_) (mm/s) | EO | *AM* | Young | 3.52 (0.73) | **F=141.930 P<0.001 η²=0.807** | **F=14.824 P<0.001 η²=0.310** | F=1.873 P=0.180 η²=0.052 | **F=6.017 P=0.020 η²=0.154** | **F=28.121 P<0.001 η²=0.453** | F=2.400 P=0.131 η²=0.068 | F=0.431 P=0.586 η²=0.013 | F=1.320 P=0.269 η²=0.038 | F=0.020 P=0.888 η²=0.001 | F=0.100 P=0.753 η²=0.003 | F=1.405 P=0.252 η²=0.040 | F=0.681 P=0.462 η²=0.020 | F=.349 P=0.637 η²=0.010 | F=0.575 P=0.582 η²=0.017 |
|  |  |  | Old | 3.76 (1.14) |  |  |  |  |  |  |  |  |  |  |  |  |  |  |
|  |  | *PM* | Young | 3.14 (0.61) |  |  |  |  |  |  |  |  |  |  |  |  |  |  |
|  |  |  | Old | 3.57 (1.29) |  |  |  |  |  |  |  |  |  |  |  |  |  |  |
|  | EC | *AM* | Young | 4.06 (0.97) |  |  |  |  |  |  |  |  |  |  |  |  |  |  |
|  |  |  | Old | 4.90 (2.33) |  |  |  |  |  |  |  |  |  |  |  |  |  |  |
|  |  | *PM* | Young | 3.61 (0.91) |  |  |  |  |  |  |  |  |  |  |  |  |  |  |
|  |  |  | Old | 4.30 (1.93) |  |  |  |  |  |  |  |  |  |  |  |  |  |  |
|  | Unstable | *AM* | Young | 7.55 (1.93) |  |  |  |  |  |  |  |  |  |  |  |  |  |  |
|  |  |  | Old | 8.36 (2.54) |  |  |  |  |  |  |  |  |  |  |  |  |  |  |
|  |  | *PM* | Young | 6.94 (2.07) |  |  |  |  |  |  |  |  |  |  |  |  |  |  |
|  |  |  | Old | 7.63 (2.35) |  |  |  |  |  |  |  |  |  |  |  |  |  |  |
| RMS (COP _AP_) (mm) | EO | *AM* | Young | 3.22 (1.00) | **F=89.224 P<0.001 η²=0.724** | **F=15.025 P<0.001 η²=0.313** | **F=5.877 P=0.021 η²=0.147** | F=2.776 P=0.105 η²=0.078 | F=0.802 P=0.377 η²=0.023 | F=0.006 P=0.9411 η²=0.000 | F=0.138 P=0.841 η²=0.004 | F=0.986 P=0.378 η²=0.029 | F=0.062 P=0.805 η²=0.002 | F=0.041 P=0.840 η²=0.001 | F=0.023 P=0.978 η²=0.001 | F=0.180 P=0.836 η²=0.005 | F=0.376 P=0.688 η²=0.011 | F=0.360 P=0.699 η²=0.011 |
|  |  |  | Old | 4.23 (1.60) |  |  |  |  |  |  |  |  |  |  |  |  |  |  |
|  |  | *PM* | Young | 3.41 (1.07) |  |  |  |  |  |  |  |  |  |  |  |  |  |  |
|  |  |  | Old | 4.23 (1.47) |  |  |  |  |  |  |  |  |  |  |  |  |  |  |
|  | EC | *AM* | Young | 3.89 (1.11) |  |  |  |  |  |  |  |  |  |  |  |  |  |  |
|  |  |  | Old | 4.63 (1.35) |  |  |  |  |  |  |  |  |  |  |  |  |  |  |
|  |  | *PM* | Young | 3.92 (1.54) |  |  |  |  |  |  |  |  |  |  |  |  |  |  |
|  |  |  | Old | 4.88 (1.60) |  |  |  |  |  |  |  |  |  |  |  |  |  |  |
|  | Unstable | *AM* | Young | 5.94 (1.79) |  |  |  |  |  |  |  |  |  |  |  |  |  |  |
|  |  |  | Old | 6.93 (1.66) |  |  |  |  |  |  |  |  |  |  |  |  |  |  |
|  |  | *PM* | Young | 6.00 (1.94) |  |  |  |  |  |  |  |  |  |  |  |  |  |  |
|  |  |  | Old | 7.17 (1.82) |  |  |  |  |  |  |  |  |  |  |  |  |  |  |
| RMS (COP _ML_) (mm) | EO | *AM* | Young | 1.52 (0.52) | **F=207.011 P<0.001 η²=0.859** | **F=12.484 P<0.001 η²=0.274** | F=0.542 P=0.467 η²=0.016 | F=0.688 P=0.413 η²=0.020 | F=0.251 P=0.619 η²=0.007 | F=0.102 P=0.751 η²=0.003 | F=0.017 P=0.935 η²=0.000 | F=0.126 P=0.778 η²=0.004 | F=0.084 P=0.773 η²=0.002 | F=0.040 P=0.844 η²=0.001 | F=0.569 P=0.500 η²=0.016 | F=0.232 P=0.695 η²=0.007 | F=0.133 P=0.785 η²=0.004 | F=0.027 P=0.921 η²=0.001 |
|  |  |  | Old | 1.66 (0.69) |  |  |  |  |  |  |  |  |  |  |  |  |  |  |
|  |  | *PM* | Young | 1.54 (0.66) |  |  |  |  |  |  |  |  |  |  |  |  |  |  |
|  |  |  | Old | 1.76 (0.78) |  |  |  |  |  |  |  |  |  |  |  |  |  |  |
|  | EC | *AM* | Young | 1.67 (0.84) |  |  |  |  |  |  |  |  |  |  |  |  |  |  |
|  |  |  | Old | 1.98 (1.07) |  |  |  |  |  |  |  |  |  |  |  |  |  |  |
|  |  | *PM* | Young | 1.66 (0.73) |  |  |  |  |  |  |  |  |  |  |  |  |  |  |
|  |  |  | Old | 1.81 (0.89) |  |  |  |  |  |  |  |  |  |  |  |  |  |  |
|  | Unstable | *AM* | Young | 4.11 (0.93) |  |  |  |  |  |  |  |  |  |  |  |  |  |  |
|  |  |  | Old | 4.32 (1.34) |  |  |  |  |  |  |  |  |  |  |  |  |  |  |
|  |  | *PM* | Young | 4.27 (1.57) |  |  |  |  |  |  |  |  |  |  |  |  |  |  |
|  |  |  | Old | 4.44 (1.84) |  |  |  |  |  |  |  |  |  |  |  |  |  |  |
